# Untying Moral Efficacy and Meaningfulness in Promoting Students’ Social Entrepreneurial Intentions: The Mediating Role of Positive Reciprocity

**DOI:** 10.3389/fpsyg.2022.915725

**Published:** 2022-05-09

**Authors:** Jian Xiang, Yanjun Zhang

**Affiliations:** ^1^Training Centre, The United Front Work Department of CPC Central Committee, Beijing, China; ^2^College of Chinese Language and Culture, Jinan University, Guangzhou, China

**Keywords:** entrepreneurship intentions, moral efficacy, moral meaningfulness, positive reciprocity, SEM

## Abstract

This study chooses to describe social entrepreneurship as a social mission that enables business students to identify opportunities to launch start-ups and social enterprises by understanding the nature of social responsibility and fostering a reciprocal attitude to solve social issues. We collected data on students from different business schools in mainland China through a structured questionnaire (*n* = 326) and analyzed them through structural equation modeling (SEM). The results show that moral efficacy and meaningfulness are positively related to positive reciprocity, which leads to students’ social entrepreneurial intentions. The study concluded that potential social entrepreneurs should take ethical courses as part of their training to help them develop a responsible mentality and social entrepreneurial aspirations. On this basis, the practical and theoretical significance of this study is proposed, and its limitations and future development directions are pointed out.

## Introduction

Social entrepreneurship creates a positive impact on societies through the social mission of identifying opportunities and initiating business startups (Jiatong et al., 2021). But the existing academic practices do not incorporate effective mechanisms and exact content information to enable students to find social market opportunities and launch social enterprises (Douglas, 2015; Hockerts, 2018). Social entrepreneurship is still at its infancy level where business schools have merely begun to motivate their students to pursue a career as social entrepreneurs (Smith et al., 2007; Miller et al., 2012; Choi and Majumdar, 2014; Hjorth and Holt, 2016). Therefore, business schools need to design social entrepreneurship courses in a way that morally motivates students and gives them some ethical meaning for appreciating the benefits of social entrepreneurship and developing a reciprocal attitude toward social responsibility (Zhou et al., 2021). This sense of responsibility could be aroused by explaining the significance of addressing social issues rather than the courses changing students’ intentions (Hockerts, 2018). By doing this, the authors aim to address the empirical gap in the syllabi and coursework of social entrepreneurship in the existing educational system in business schools (Miller et al., 2012; Cooper and Greene, 2016; Zhu et al., 2016; Kickul et al., 2018; Steiner et al., 2018).

Current research opts to explore whether students’ participation in social entrepreneurship courses changes their mindset to consider it as a career path or there should be some extracurricular activities arising their moral efficacy and meaningfulness which could result in a reciprocal attitude and ultimately into social entrepreneurship intentions. It is needless to mention how important it is for students to study social entrepreneurship as a career path and develop a positive reciprocal attitude to address social issues and contribute to society (Zhang et al., 2014; Fayolle and Gailly, 2015). However, relevant literature is scarce because only a couple of studies have been carried out to test the impact of social entrepreneurship education in business schools. For example (Chang et al., 2014), in their study about entrepreneurship modules in undergraduates argued that pedagogical methods should incorporate projects that could generate income and as well as increase students’ awareness, communication, and empathy by leading them toward novel ideas. The outcomes of this study are consistent with the findings of Kwong et al. (2012) who claimed that such teaching mechanisms aware students of social and civic issues and arouse positive reciprocity to contribute. The study of Kwong et al. (2022) argued that social entrepreneurial courses could flourish students’ aspirations to involve in social entrepreneurial behaviors. This agrees with the conclusions of Shah et al. (2022) that moral obligation among students would shape their positive reciprocal attitude to help marginalized people. Henceforth, authors assume that if business schools appropriately organize and structure mechanisms to engage their students in social entrepreneurial initiatives they can probably come up with social value creation (Chavali et al., 2022). On this basis, this study chooses to answer the following research questions. (1) How do moral efficacy and meaningfulness promote students’ social entrepreneurship intention? (2) What is the role of positive reciprocity in the formation of students’ social entrepreneurship intention.

The remainder of the article deals with the theoretical framework and development of hypotheses in section 2. Section 3 deals with the methodology adopted for current research. Section 4 interprets results derived from statistical analysis. And section 5 discusses findings, implications to theory and practice, including limitations, and recommendations for future research.

## Theoretical Foundation and Development of Hypotheses

Traditional theories about cognitive learning recommend teachers develop comprehension among students regarding definitions, frameworks, and theories, unlike existing pedagogies which merely act as a deposit of knowledge (Hockerts, 2018). Learning needs to be a cognitive as well as a social process including interdependent actions that could shape students’ social interactive dynamics (Branzei and Fredette, 2008; Tuan and Pham, 2022). Theories on social learning have substantially marked their impact on the management of social entrepreneurship classrooms by introducing experiential learning that comes from interacting with teachers, co-learners, and industry experts (Kolb and Kolb, 2005; Kickul et al., 2012; Sharahiley, 2020). According to the findings Kolb and Kolb (2005), experiential learning could motivate students to take part in conversational learning which would substantially develop their expertise in social entrepreneurship. Another study by Barry and Meisiek (2015) explored that studio teaching would rather enhance materiality and invention among students as compared to traditional classroom teaching (Douglas, 2015) to involve in field-based research about diverse social problems and explore potential opportunities for start-ups by themselves.

**FIGURE 1:**
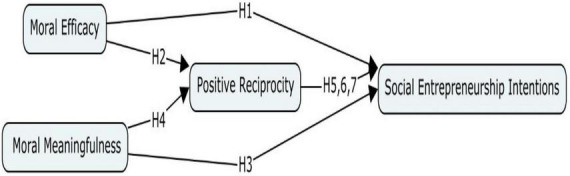
Theoretical framework.

The study explores how moral efficacy through social entrepreneurship self-motivators could explore various opportunities to contribute by satisfying social needs (Zahra et al., 2009). Besides, such moral meaningfulness could be formed among students through diverse interactions (Bandura, 1991; Treviño et al., 2006) and the identification of entrepreneurship opportunities (Bird, 1988) that could trigger positive reciprocity among students toward social entrepreneurship intentions (Hashim et al., 2018; Chavali et al., 2022). For detailed hypothesized relations refer Figure 1.

### Moral Efficacy

Moral efficacy defines individuals’ belief in their capability to face the probable ethical issues in personal and professional lives such as the ability to tackle ethical dilemmas by providing ethical solutions (Hannah et al., 2011). Moral efficacy showcases individuals’ attitudes to foster positive and ethical resources. According to Desplaces et al. (2007) developing moral competencies in ethical decision-making nurtures individuals’ potential in fixing issues on ethical grounds. Similarly, May et al. (2003) argued that if the leaders can convert intentions into ethical actions in conflict situations this could further enhance their moral efficacy at a substantial level. Simultaneously, Kwong et al. (2022) in their study about university’s role and potential of social entrepreneurial elective courses found that social entrepreneurial courses widen students’ awareness and nurture their aspirations to indulge in social entrepreneurial behaviors. However, in terms of goals and their implementation toward success business ethics may vary from case to case to the extent they provide a psychologically safe environment, minimize the threat, and retain motivation to face diverse challenges of ethical dilemmas. Recent studies have established that going through such experiences considerably enhances moral efficacy (Hannah et al., 2011; Nelson et al., 2012) which in turn adds value to the community through the creative use of the resources either in starting a new business or transforming an existing one (Shaw and Carter, 2007; Bosch-Badia et al., 2015; Tuan and Pham, 2022). Through social entrepreneurship, self-motivators explore various opportunities to contribute by making social changes or adding value to society’s needs (Zahra et al., 2009). We argue that when individuals perceive a win-win situation they tend to practice reciprocity by adaptation and knowledge transfer between the parties involved (Jackson and Sparks, 2005) which positively influences the relationship quality, performance, and business negotiations (Hoppner et al., 2015; Arnesen and Foster, 2018; Shahzad et al., 2021). On the basis of aforementioned rationale, we posit following hypotheses:

H1: Moral efficacy will promote students’ intentions for social entrepreneurship.

H2: Moral efficacy will promote students’ positive reciprocity toward intentions for social entrepreneurship.

### Moral Meaningfulness

Naturally, individuals possess a motive to seek meaning in their respective lives which can be categorized differently such as a sense of goodness or positivity to life or self-belief that enables people to understand their reciprocal attitude that can make a social difference (Baumeister and Vohs, 2002). According to the findings of Aktouf (1992) and Wrzesniewski et al. (1997) lack of meaningfulness in life can lead to disengagement therefore, being meaningful in life keeps up motivation and gives reason and strength to deal with the moral dilemmas (May et al., 2014). Similarly, Lukman et al. (2021) in their study about diaspora social entrepreneurship intentions explored that social responsibility and service learning substantially developed social entrepreneurial attitude among Ghanaian students. Earlier research has established that such moral meaningfulness could be formed among students through diverse interactions (Bandura, 1991; Treviño et al., 2006) and the identification of entrepreneurship opportunities (Bird, 1988) henceforth, students must be able to comprehend and predict social entrepreneurship behaviors (Krueger et al., 2000; Kolvereid and Isaksen, 2006). We argue that if the students are given classroom discussions and group case analyses including paper assignments focusing on ethical issues. Such practices would help students to comprehend the significance of moral values and traits. Moreover, their conceptualization of moral meaningfulness could give them a clear understanding of its role in their personal and professional identity and trigger a positive reciprocal attitude thereon. Based on the rationale, we posit the following hypotheses:

H3: Moral meaningfulness will promote students’ intentions for social entrepreneurship.

H4: Moral meaningfulness will promote students’ positive reciprocity toward intentions for social entrepreneurship.

### Positive Reciprocity

The study by Gouldner (1960) defined reciprocity as a mutual exchange of benefits among units which is further elaborated by Gergen (1969) like repayment of good/bad deeds among individuals, groups, or societies. Naturally, when one party benefits the other a moral obligation is generated to return the favor. The parties involved maintain homeomorphic equivalence of reciprocity through repaying positive actions for positive actions and negative actions for negative actions (Cropanzano et al., 2017; Evans et al., 2019). Since social entrepreneurship primarily incorporates business and social welfare, therefore, it is mandatory to develop spiritual and immaterial relations among individuals and groups (Pache and Santos, 2013) so that a norm of positive reciprocity could be established by giving credit to others for what they have accomplished (Gouldner, 1960). Consequently, this could trigger positive reciprocity to repay the good deeds. According to the findings of Shah et al. (2022) moral obligation among students could shape their positive reciprocal attitude to help marginalized people. Recent study carried out by Chavali et al. (2022) found that if business schools had well organized and structured mechanism to engage students in social entrepreneurial initiatives it could lead them toward social value creation. Hashim et al. (2018) in their study about digital piracy established significant mediating potential of moral obligation between attitudes, subjective norms and intentions. Another study by Rivis et al. (2009) also similarly argued that moral norms predict positive reciprocal attitudes and obligations which is congruent with the findings of Beck and Ajzen (1991) and Cronan and Al-Rafee (2008). This is consistent with the findings of Cropanzano et al. (2017) who explored that good deeds generate positive reciprocal attitudes. Henceforth, positive reciprocal attitude among parties could further strengthen their commitment and trust because positive reciprocity creates a strong bond among the parties (André et al., 2017). On the basis of aforementioned rationale we posit following hypotheses:

H5: Positive reciprocity will promote students’ intentions for social entrepreneurship.

H6: Positive reciprocity mediates positive relation between students’ moral efficacy and social entrepreneurship intentions.

H7: Positive reciprocity mediates positive relation between students’ moral meaningfulness and social entrepreneurship intentions.

## Research Methodology

The purpose of the current study was to emphasize antecedents that added value to the students’ moral efficacy and meaningfulness to enhance positive reciprocity which ultimately resulted in their social entrepreneurship intentions. Therefore, we only targeted business school students in central parts of China by using the convenience sampling method. We distributed 500 questionnaires via WeChat groups, QQ groups, and email addresses. The questionnaire started with an introductory paragraph explaining the survey’s purpose. Survey respondents were above the age of 18 years and studying in different business schools in China. It took us 4 weeks to gather the data. Three hundred thirty students responded to our questionnaire out of which 04 responses were incomplete henceforth, they were deleted from the dataset. The survey was responded to with a considerably high rate of 66 percent. When there is a high response to any survey it ensures that non-response bias is at its minimum level.

### Instrument Validity

The survey questionnaire contained 24 items in total and two sections. Section no. 1 inquired about demographic information about respondents, Table 1 provides detailed information about respondents. The second section of the survey questionnaire incorporated items to assess participants’ social entrepreneurship intentions based on the studied factors. The item scales for moral efficacy were adapted from Parker (1998) and May et al. (2014) but we deleted one item (ME4) due to its meager factor loading, for moral meaningfulness (May et al., 2014), and for positive reciprocity, we adapted item scales from Caliendo et al. (2012). However, we adapted item scales for social entrepreneurship intentions among students from Defourny and Kim (2011).

**TABLE 1:** The demographic characteristics of the sample.

	Frequency	% age
**Gender**		
Male	173	53
Female	153	47
Total	326	100.0
**Age**		
18–20	125	38
21–22	130	40
23–24	71	22
Total	326	100.0
**Education**		
Undergraduate	150	46
Graduates/Postgraduate	176	54
Total	326	100.0

## Results

### Reliability and Convergent Validity

We analyzed data by using a two-step approach. In the first step, the measurement model was assessed to estimate the structural model and hypothetical relationships by evaluating the framework’s predictive capability, followed by the evaluation of factor loadings which determined reliability among the item scales. The average variance extracted (AVE) was also examined to determine convergent liability. Later, composite reliability (CR) and Cronbach’s alpha (CA) were calculated to determine internal consistency among item scales. For detailed main statistics refer to Table 2.

**TABLE 2:** Main statistics.

Factor	Item	Factor loading	AVE	rhoA	Alpha
Moral efficacy	ME1	0.827	0.684	0.946	0.945
	ME2	0.819			
	ME3	0.788			
	ME5	0.857			
	ME6	0.842			
	ME7	0.789			
	ME8	0.855			
	ME9	0.834			
Moral meaningfulness	MM1	0.784	0.668	0.891	0.890
	MM2	0.790			
	MM3	0.860			
	MM4	0.834			
Positive reciprocity	PR1	0.718	0.600	0.821	0.817
	PR2	0.792			
	PR3	0.811			
Social entrepreneurship intentions	SEI1	0.804	0.668	0.943	0.941
	SEI2	0.815			
	SEI3	0.761			
	SEI4	0.871			
	SEI5	0.849			
	SEI6	0.728			
	SEI7	0.891			
	SEI8	0.807			

Variance inflated factor (VIF) was utilized to assess multicollinearity issues. The statistical results obtained are congruent with standard thresholds recommended by Hair et al. (2011). The highest stats found in our results represent VIF value of (1.384) which is substantially lower than the suggested threshold and therefore, does not denote any concerns for multicollinearity. Table 3 represents overall achieved VIF stats.

**TABLE 3:** Inner VIF values.

Exogenous latent variable	VIF
Moral efficacy	1.384
Moral meaningfulness	1.347
Positive reciprocity	1.295

### Discriminant Validity

In Table 4, we have opted to compare the square roots of the AVE with correlation coefficients to determine discriminant validity where all the factors studied were found to be stronger in comparison to the corresponding correlation coefficients (Fornell and Larcker, 1981; Barclay et al., 1995).

**TABLE 4:** Discriminant validity.

	ME	MM	PR	SEI
Moral efficacy	**0.827**			
Moral meaningfulness	0.457	**0.818**		
Positive reciprocity	0.421	0.393	**0.775**	
Social entrepreneurship intentions	0.619	0.478	0.619	**0.817**

*The bolded value indicates square root AVE.*

### Hypotheses Test

Guidelines from Henseler et al. (2009) were followed for measuring the significance and effect of path coefficients. To achieve the purpose, a bootstrapping mechanism was adopted in SmartPLS with 5,000 samples (Hair et al., 2011).

**FIGURE 2:**
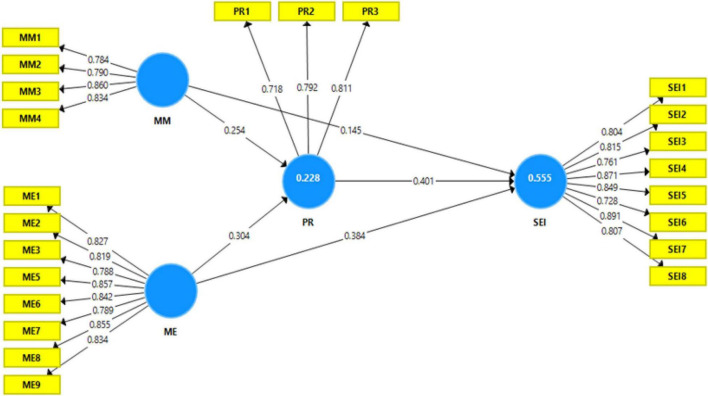
Structural model results.

The obtained stats empirically support overall hypothesized relations. Further details are provided in Figure 2 and Table 5.

**TABLE 5:** Path coefficients and significances.

							Confidence intervals	
	Relationship	Beta	STDEV	*t*-value	*P-*values	Effect size	2.5%	97.5%
H1	Moral efficacy -> SEI	0.384	0.069	5.592	0.000	0.239	0.237	0.505
H2	Moral efficacy -> PR	0.304	0.067	4.521	0.000	0.095	0.169	0.431
H3	Moral meaningfulness -> SEI	0.145	0.060	2.411	0.016	0.035	0.024	0.257
H4	Moral meaningfulness -> PR	0.254	0.071	3.573	0.000	0.066	0.112	0.389
H5	Positive reciprocity -> SEI	0.401	0.056	7.117	0.000	0.278	0.289	0.510
H6	ME -> PR -> SEI	0.122	0.033	3.692	0.000			
H7	MM -> PR -> SEI	0.102	0.033	3.092	0.002			

### The Predictive Power of the Model

The evaluation of *R*^2^ in SmartPLS 3 confirmed the framework’s prediction capability. We utilized PLS-Algorithm to calculate *R*^2^ and the stats achieved represent *r*-squared values of (0.228) for positive reciprocity, and (0.555) for social entrepreneurship intentions. As suggested by Falk and Miller (1992) *r*-square values that exceed (0.010) could be accepted in social sciences research. Accordingly, the results extracted in our study surpass the considerable threshold suggested.

Lastly, for measuring endogenous constructs’ weight *Q*^2^ we assessed through a blindfolding procedure which determined cross-validated redundancy (Fornell, 1994). Chin (1998) recommended that if Q^2^ stats for endogenous constructs tend to be higher than zero, then the framework showcases predictive relevance. The results for positive reciprocity (0.123) and social entrepreneurship intentions (0.339) are above the threshold suggested. Henceforward, the studied framework denotes substantial predictive relevance.

## Discussion of Findings

Based on social learning theory, this study makes an important contribution to the existing literature on social entrepreneurial intentions by complementing the reciprocity attitudes of business school students in creating value for society or converting job seekers into social entrepreneurs. This comprehensive research model works well and adds value to further understanding of social entrepreneurship factors in the Chinese context. Statistical results support all assumptions and provide valuable insights.

Drawing upon the confirmed hypotheses, the current study concludes that social entrepreneurship education significantly and positively affects students’ intentions to start a social enterprise where students get the purposeful motivation to reciprocate their contribution to society. This is consistent with the findings of Smith and Woodworth (2012) that social entrepreneurship education could help students improve social entrepreneurship self-efficacy. The findings further explain that with enhanced self-efficacy students tend to derive moral values and meaningfulness within their self-efficacy which is in line with the arguments of Chang et al. (2014) that experiential learning on social entrepreneurship affects students’ characteristics to reciprocate accordingly. Henceforth, the findings emphasize the inclusion of a social business plan as a pedagogical tool to lead students toward getting on with their respective social enterprises. Our findings further explained that the success of the social entrepreneurship education mechanism is in creating a moral obligation for social issues which is consistent with the conclusions of Smith et al. (2007) that social entrepreneurship education influences students’ empathy toward social issues. Similarly, such students who self-selected social entrepreneurship education could have reached a certain level of moral efficacy and meaningfulness which could result in positive reciprocity toward addressing social issues.

To address this issue pedagogical methods in business schools, need to ensure a separate mechanism to monitor the impact of core and elective courses of social entrepreneurship to differentiate between students who self-selected and randomly selected courses. This is congruent with the findings of Rasche et al. (2013) and Rasche and Gilbert (2015) who argued that the impact of social entrepreneurship education could not be much effective unless the curriculum was separated from the core business curriculum to a specific one. Moreover, if the curriculum is not decoupled then the primary purpose of the managers would be merely increasing profitability for stakeholders rather than changing their moral obligations. These findings are consistent with those (Blasco, 2012) who argued that to make the students morally and socially responsible, business schools need to modify the message within the isolated elective courses and align the meta-messages being conveyed as part of the curriculum. Such changes would play a significant role in changing students’ moral values and meaningfulness that could potentially arise their positive reciprocal attitude toward social entrepreneurship intentions.

## Conclusion and Implications

### Conclusion

This research study opts to portray social entrepreneurship as a social mission for business students to visualize potential venues for launching startups and social enterprises. Because as students begin to understand their social responsibility, they embark on fostering a reciprocal attitude to address social issues.

This study adds value to the theoretical and practical horizon of social entrepreneurship by explaining how business schools can play their role in advancing their student’s moral obligation to reciprocate positively by responding to social needs and issues. Current research has proved that pedagogical methods can significantly and positively affect students’ moral efficacy, moral meaningfulness, and positive reciprocity toward social entrepreneurship intentions. The participants in the collected data are diverse in terms of age, culture, and educational background. The statistical results are also robust, they have sufficiently evidenced the hypothesized relations and generated valuable insights for theory and practice. Finally, it is expected that future studies shall come up with more interventions like ours to explore students’ intentions and behavior because students’ ability to develop social entrepreneurship intentions depends more on the pedagogical methods practiced in the business schools rather than the course contents.

### Implications

Promoting students’ moral obligation and a positive reciprocal attitude is of utmost importance these days to arouse their social responsibility to address social issues. And many business schools are playing a significant role in developing social entrepreneurial behavior among students (Brock and Kim, 2011). Henceforth, the findings of this study recommend valuable implications for educators who intend to develop motivation and meaningfulness among their students to reciprocate positively to their society by coming up with novel ideas and social venture startups. The course instructors need to incorporate experiential learning throughout their pedagogy to develop interactive skills with classroom participants as well as with industry experts. So that students’ moral efficacy to add some value to society could be enhanced. For example, course instructors could modify their traditional teaching methods with case-based learning. The case teaching method enables students to make a SWOT analysis (strengths, weaknesses, opportunities, and threats), such a practice would rather add value to the experiential learning of their students by discussing the studied case’s overall performance, achievement, failures, weaknesses, or strength.

Likewise, studio learning could also be very effective in developing efficacy and meaning among students to nurture their social entrepreneurial intentions for addressing social issues. Outcomes through studio learning could be even greater because this methodology involves students conducting field surveys and interviews. For studio learning, students need to pay visits to various social enterprises to work firsthand about the phenomenon they are trying to explore. Such industry visits would also play a very important role to enhance interaction with prospective investors that could help students to act upon their social entrepreneurial intentions at a later stage, such a motivation can further influence students to rehearse their social entrepreneurial intentions for a more value-driven leadership (Gentile, 2012). To achieve this milestone, business schools can contribute by combining electives with the course’s meta-information. The result of such a major change will bring greater moral efficacy and meaning to business students, developing positive reciprocity and value creation for society.

### Limitations and Future Research Recommendations

While investigating factors for social entrepreneurship intentions among students our study was exposed to many limitations. First, the existing students were considered to determine their future social entrepreneurship intentions based on current course contents and pedagogical methods which might vary or change over time. Second, the statistical analysis comprises cross-sectional data which encompasses the risk of attitudinal or behavioral change during or after the studentship of the participants. Third, successful studio teaching surpasses the usual business school classroom settings and requires more resources to be invested which indicates that all the business schools may not be able to afford it. Future research is recommended to replicate our findings in other national/international universities to confirm its external validity and generalizability in a local, international, or cross-cultural context.

## Data Availability Statement

The raw data supporting the conclusions of this article will be made available by the authors, without undue reservation.

## Ethics Statement

Ethical review and approval was not required for the study on human participants in accordance with the Local Legislation and Institutional Requirements. Written informed consent for participation was not required for this study in accordance with the National Legislation and the Institutional Requirements.

## Author Contributions

JX: conceptualization, writing—original draft preparation, data curation, software, and formal analysis. YZ: supervision, methodology, fund acquisition, and writing—review and editing. Both authors contributed to the article and approved the submitted version.

## Conflict of Interest

The authors declare that the research was conducted in the absence of any commercial or financial relationships that could be construed as a potential conflict of interest.

## Publisher’s Note

All claims expressed in this article are solely those of the authors and do not necessarily represent those of their affiliated organizations, or those of the publisher, the editors and the reviewers. Any product that may be evaluated in this article, or claim that may be made by its manufacturer, is not guaranteed or endorsed by the publisher.
